# Elicitation of T-cell-derived IFN-γ-dependent immunity by highly conserved *Plasmodium ovale curtisi* Duffy binding protein domain region II (PocDBP-RII)

**DOI:** 10.1186/s13071-023-05897-9

**Published:** 2023-08-08

**Authors:** Zhenyu Ren, Qiyang Shi, Simin Xu, Jiahui Xu, Yi Yin, Zhijie Lin, Sui Xu, Xiaoqin Ma, Yaobao Liu, Guoding Zhu, Xinlong He, Jingyuan Lu, Yinyue Li, Wenwen Zhang, Jiali Liu, Yun Yang, Eun-Taek Han, Jun Cao, Feng Lu

**Affiliations:** 1 Department of Pathogenic Biology and Immunology, School of Medicine, Yangzhou University, Yangzhou, 225009 People’s Republic of China; 2 National Health Commission Key Laboratory of Parasitic Disease Control and Prevention, Jiangsu Provincial Key Laboratory On Parasite and Vector Control Technology, Jiangsu Provincial Medical Key Laboratory, Jiangsu Institute of Parasitic Diseases, Wuxi, 214064 People’s Republic of China; 3 Changshu Second People’s Hospital, Suzhou, 215500 Jiangsu People’s Republic of China; 4 Department of Medical Environmental Biology and Tropical Medicine, School of Medicine, Kangwon National University, Chuncheon, Gangwon-do 24341 Republic of Korea; 5 Affiliated Hospital of Yangzhou University, Yangzhou, 225000 People’s Republic of China; 6 Jiangsu Key Laboratory of Experimental & Translational Non-Coding RNA Research, School of Medicine, Yangzhou University, Yangzhou, 225009 People’s Republic of China

**Keywords:** *Plasmodium ovale curtisi*, *Duffy binding protein domain region II*, Genomic diversity, Antigenicity, Immunogenicity

## Abstract

**Background:**

Infections with *Plasmodium ovale* are widely distributed but rarely investigated, and the resulting burden of disease has been underestimated. *Plasmodium ovale curtisi* Duffy binding protein domain region II (*PocDBP-RII*) is an essential ligand for reticulocyte recognition and host cell invasion by *P. ovale curtisi*. However, the genomic variation, antigenicity and immunogenicity of *PocDBP-RII* remain major knowledge gaps.

**Methods:**

A total of 93 *P. ovale curtisi* samples were collected from migrant workers who returned to China from 17 countries in Africa between 2012 and 2016. The genetic polymorphism, natural selection and copy number variation (CNV) were investigated by sequencing and real-time PCR. The antigenicity and immunogenicity of the recombinant PocDBP-RII (rPocDBP-RII) protein were further examined, and the humoral and cellular responses of immunized mice were assessed using protein microarrays and flow cytometry.

**Results:**

Efficiently expressed and purified rPocDBP-RII (39 kDa) was successfully used as an antigen for immunization in mice. The haplotype diversity (Hd) of *PocDBP-RII* gene was 0.105, and the nucleotide diversity index (π) was 0.00011. No increased copy number was found among the collected isolates of *P. ovale curtisi*. Furthermore, rPocDBP-RII induced persistent antigen-specific antibody production with a serum IgG antibody titer of 1: 16,000. IFN-γ-producing T cells, rather than IL-10-producing cells, were activated in response to the stimulation of rPocDBP-RII. Compared to PBS-immunized mice (negative control), there was a higher percentage of CD4^+^CD44^high^CD62L^−^ T cells (effector memory T cells) and CD8^+^CD44^high^CD62L^+^ T cells (central memory T cells) in rPocDBP-RII‑immunized mice.

**Conclusions:**

*PocDBP-RII* sequences were highly conserved in clinical isolates of *P. ovale curtisi*. rPocDBP-RII protein could mediate protective blood-stage immunity through IFN-γ-producing CD4^+^ and CD8^+^ T cells and memory T cells, in addition to inducing specific antibodies. Our results suggested that rPocDBP-RII protein has potential as a vaccine candidate to provide assessment and guidance for malaria control and elimination activities.

**Graphical Abstract:**

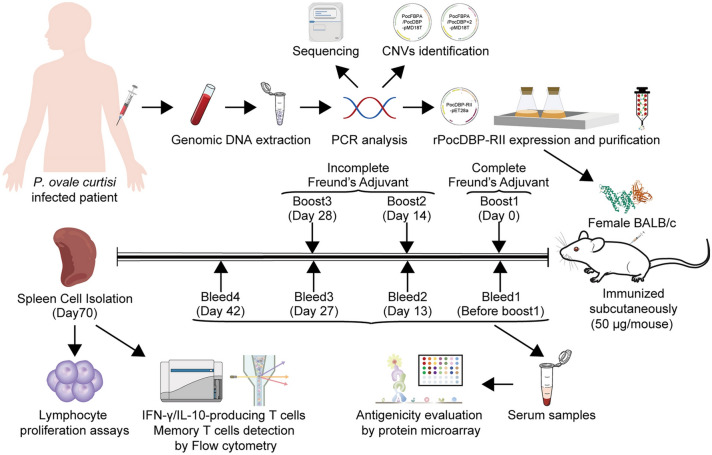

## Background

Malaria is a disease caused by *Plasmodium* species, and there were an estimated 247 million malaria cases in 2021 globally, with most of the increase in cases over the past 5 years occurring in the WHO African Region [1]. Among the five species of malaria parasites infecting humans, *Plasmodium ovale* is often overlooked because of the benign symptoms of malaria it causes. *Plasmodium ovale* has proven to be divided into two genetically distinct subspecies called *P. ovale curtisi* and *P. ovale wallikeri* [2], with the former causing a higher proportion of imported cases, a longer incubation period and higher platelet counts and total leukocyte counts than malaria caused by the latter [3, 4]. However, the morphological characteristics, clinical syndrome, response to therapy and relapsing features of *P. ovale* are similar to those of *Plasmodium vivax* [5], making it easy to be mistaken for *P. vivax* in routine diagnosis and therefore underestimating the worldwide prevalence of *P. ovale* [3, 6]. For example, a descriptive study showed that imported *P. ovale* from Africa accounted for a large proportion of total malaria cases in Henan Province, China, even more than that of *P. vivax* [7]. Malaria elimination programs in Africa therefore need to include strategies to control these hitherto neglected malaria parasites.

Duffy-binding protein (DBP) is considered a promising blood-stage vaccine candidate [8] that is involved in the invasion of host reticulocytes by merozoites of *Plasmodium vivax* and *P. knowlesi* through interaction with the host chemokine Duffy antigen receptors (DARCs) [9, 10]. The functional receptor-binding domains of PvDBP have been traced to the N-terminal cysteine-rich region II (DBP-RII), which also known as the Duffy-binding-like (DBL) domain [11]. Nevertheless, under immune selective pressure, the allelic diversity may lead to loss of reactivity and functional immunogenicity of PvDBP-RII, which is a potential challenge for vaccine development [12, 13]. Another important type of genomic variation in *Plasmodium* is copy number variation (CNV) [14]. Indeed, single nucleotide polymorphisms (SNPs) [15] and CNVs [16] have been identified in *PvDBP-RII*, which could promote evasion of *P. vivax* against PvDBP humoral immunity, limiting the efficacy of the vaccine. Specifically, functional antibodies against DBP show target priority epitopes [17], but allelic variants of DBPII can alter the reactivity of strain-specific antibodies other than MBC-derived antibodies [18]. Furthermore, data from mouse models of malaria infection suggest that CD4^+^ and CD8^+^ T cells may control the production of cytokines such as IFN-γ and IL-10 [19], and then memory T cells mediate protective blood-stage immunity [20, 21].

A previous study reported that *P. ovale curtisi* Duffy binding protein domain region II (PocDBP-RII) may act as an important ligand in the invasion process of reticulocytes by *P. ovale curtisi* [22]. However, previous studies have not been devoted to uncovering the genetic diversity and gene amplification of *PocDBP-RII*. In this study, we selected 93 *P. ovale curtisi* isolates from malaria-endemic areas in Africa during 2012–2016 to investigate the genetic polymorphism, natural selection and CNVs of *PocDBP-RII.* In addition, to investigate new information on the suitability of rPocDBP-RII as a novel vaccine candidate for *P. ovale curtisi*, the antigenicity and immunogenicity of rPocDBP-RII were measured by protein microarrays and flow cytometric analysis.

## Methods

### Genomic DNA extraction, sequencing and sequence data collection

A total of 93 blood samples were obtained from imported malaria patients with *P. ovale* identified by microscopic and epidemiological investigation in a malaria diagnostic reference laboratory in Jiangsu Province, China, from 2012 to 2016. The *Plasmodium* genomic DNA (gDNA) from 200 μl blood samples was extracted using QIAamp^™^ DNA Blood Midi Kit (QIAGEN, Inc., Valencia, CA), according to the manufacturer’s instructions. The subspecies of *P. ovale curtisi* were further determined by nested PCR assay and real-time TaqMan PCR as reported in a previous publication [23]. The PCR products were sequenced by Tanlen-bio Scientific (Wuxi, China). The primers used for sequencing are shown in Table 1. The acquired DNA sequences were aligned with reference gene sequences taken from *PocDBP* (PlasmoDB, PocGH01_00129200) in the Plasmodium Genome Resource database.

**Table 1:** List of primers used in this study

Name	Sequence (5’ → 3’)	Length (bp)	Function
PocDBP 1F	GCTGATAAATTTGTTGTTAGGTCTGA	26	sequencing
PocDBP S1F	GCAATGCTATTCCCAATTAAAG	22	
PocDBP 1R	CCTTGACTGTTTAAATTTAAAGCATTT	27	
PocDBP S1R	CTTTAATTGGGAATAGCATTGC	22	
PocDBP-Q2’-F	ACACTGCGAAAATAATGGACTGCT	24	Construct reference plasmid with one copy of *PocDBP-RII*
PocDBP-Q2’-R2	CCATAATTAGGAACATCTCTAAAAGC	26	
PocFBPA-Q1’-EcoRI-F	*catgattacgaattc*ATGCAGGCTCCTGGTTCAGAAT	37	
PocFBPA-Q1’-BamHI-R2	*atctctagaggatcc*TTGTGTGTGCCCCATCTGCT	35	
RII/FBPA-18T-LEFT	AACCCTGGCGTTACCCAA	18	Construct reference plasmid with two copies of *PocDBP-RII*
RII/FBPA-18T-RIGHT	TTCCCAGTCACGACGTTG	18	
RII-LEFT-F	*ttgggtaacgccagggtt*ACACTGCGAAAATAATGGACTGCT	42	
RII-RIGHT-R	*caacgtcgtgactgggaa*CCATAATTAGGAACATCTCTAAAAGC	44	
PocDBP-Q2-F	AGTGACAACGATCCGAACAA	20	qPCR
PocDBP-Q2-R	ACTCAACTCTTCATCATCTGCT	22	
PocFBPA-Q1-F	GCAGGCTCCTGGTTCAGAAT	20	
PocFBPA-Q1-R	GTCTGCGTAGATTCGTCAGC	20	
PocDBP-RII-BamHI-F	tgggtcgcggatccAATATTACAAACAATGATGTAAATTATGT	44	Recombinant protein expression
PocDBP-RII-XhoI-R	*gtggtggtgctcgag*TTTTATTCCTTTCTGCGCG	34	

The restriction enzymes are indicated as italicized and underlined letters

### Nucleotide diversity and natural selection

The *PocDBP* (PlasmoDB, PocGH01_00129200) sequence was used as a template to evaluate nucleotide diversity. The multiple sequence alignments of 93 isolates containing the wild reference sequence of *PocDBP-RII* were obtained using the MUSCLE in the MEGA v7.0.18 program [24]. The number of single-nucleotide polymorphism sites (SNPs), haplotypes (H), haplotype diversity (Hd) and nucleotide diversity (π) were calculated to assess genetic diversity using the DNASP v5.10.01 program. Intra-species tests for evidence of neutrality (Tajima’s D test, Fu and Li’s D* and Fu and Li’s F* tests) were performed to determine the presence of directional or balancing selection [25, 26]. Significant positive value of Tajima’s D or Fu and Li’s D* and Fu and Li’s F* represent population contraction due to selection while negative values represent population expansion and excess of singletons. *P* < 0.05 was considered significant.

### Copy number variations (CNVs) identification in PocDBP-RII gene

The *PocDBP-RII* gene copy number (CN) was estimated by a quantitative real-time SYBR Green PCR (qPCR) assay [23, 27]. Primer sequences used for the construction of reference plasmids with one or two copies of *PocDBP-RII* are presented in Table 1. Owing to the absence of the existence of *P. ovale curtisi* isolates with known copies of the studied genes as calibrator, the reference plasmids with one copy of *PocDBP-RII* (pREF1) and two copies of *PocDBP-RII* (pREF2) were constructed by the insertion of *PocDBP-RII* (nt, 48–545) and *PocFBPA* (nt, 1–619) fragments in a ratio of 1:1 or 2:1 into the pMD18-T Vector (catalog no. 6011, TAKARA) using TA Cloning and Seamless Cloning Kit (catalog no. D7010S, Beyotime, Shanghai, China). In brief, PCR reactions were performed on an Applied Biosystems 7500 Real-Time PCR System (Applied Biosystems, USA). Each reaction consisted of 1 × ChamQ SYBR qPCR Master Mix (catalog no. Q311-02, Vazyme, Jiangsu, China), 150 nM of each forward and reverse primer, 1 × ROX Reference Dye 2, 1.2 μl of gDNA or 2 μl diluted plasmid DNA, and water up to 20 μl. The reaction conditions were 95 °C for 30 s, then 40 cycles of 95 °C for 10 s and 60 °C for 34 s. At the end of the amplification cycle, a melting curve analysis was performed to confirm that the synthesized products were correct. To exclude the effect of human gDNA extracted from whole blood, gDNA from healthy individuals was used as a negative control. At each experiment, each individual sample was run in two wells, and all samples were repeated if standard deviation (SD) of the two-threshold cycle (Ct) values was > 0.5 or ΔCt value between test sample and negative control was < 3. Text reports containing the Ct values for each well were exported to Excel (Microsoft) and analyzed by 2^−∆∆Ct^ method of relative quantification to estimate copy numbers. If the N-fold copy number was between these values (0.7 < N-fold < 1.3), the test sample was considered to carry one copy of the target gene. The test sample containing two CNs was repeated at N-fold > 1.3 to ensure the reliability of the amplification results.

### Recombinant protein expression and purification

rPocDBP-RII protein was expressed as described in a previous study [22]. Briefly, PCR amplification of *PocDBP-RII* was performed with Phusion high-fidelity DNA polymerase (catalog no. F530S, Thermo scientific, Sweden) using forward and reverse primers (Table 1) and cloned to the pET28a vector by Seamless Cloning Kit. Then, *Escherichia coli* strain Rosetta (DE3) (catalog no. CB108, TIANGEN, Beijing, China) was transformed with the recombinant plasmid, plate on a LB agar plate (containing 20 µg/ml kanamycin) and incubated overnight at 37 °C. A single colony was picked and inoculated into a 50-ml conical tube containing 20 ml LB media (supplemented with 20 µg/ml kanamycin) at 37 °C overnight with shaking at 200 rpm. The starter culture was inoculated into a flask containing 1000 ml LB media with kanamycin at 37 °C until the optical density reached 0.6 at 600 nm (OD_600_). The expression was inducted with 0.5 mM isopropyl β-D-1-thiogalactopyranoside (IPTG) (catalog no. I8070, Solarbio, Beijing, China) at 30 °C. Six hours later, the cells were harvested by centrifugation at 5000 × g for 10 min at 4 °C. The pellet was immediately stored at − 20 °C overnight.

The cells were thawed and resuspended in lysis buffer: 100 mM Na_2_HPO_4_, 10 mM Tris–HCl, 8 M urea, pH 8.0. Isolation of inclusion bodies was taken by ultrasound and centrifugation at low temperature. The washed inclusion bodies were solubilized with lysis buffer containing 8 M urea with reciprocal rotation overnight at 4 °C. rPocDBP-RII protein was purified using a high-affinity nickel-charged nitrilotriacetic acid (Ni–NTA) resin (catalog no. L00250, GenScript, Jiangsu, China). For stepwise dialysis, 6 M, 4 M and 2 M urea was utilized separately to slowly remove urea and promote refolding. Each time the sample was incubated for > 12 h at 4 °C with agitation. Ultrafiltration method was used to achieve the effect of concentration after the last time of dialysis. The purified and concentrated rPocDBP-RII protein was treated with 2 × loading dyes with and without dithiothreitol (DTT, reducing condition) and then analyzed by 12% SDS-PAGE with Coomassie Brilliant Blue R-250 PAGE staining, western blot and Pierce BCA Protein Assay Kit. In addition, the endotoxin test showed that the amount of endotoxin in rPocDBP-RII was < 1 EU/ml.

### Immunizations of mice with rPocDBP-RII

BALB/c mice (female, 6 weeks old) were obtained from the Experimental Animal Center of Yangzhou University (Yangzhou, China). Each mouse was injected subcutaneously with the emulsion containing 50 μg of rPocDBP-RII (*n* = 5) or phosphate-buffered saline (PBS; *n* = 3) mixed with Freund’s complete adjuvant (catalog no. F5881, Sigma) as the primary immunization [28, 29]. Subsequent boosts were performed with the same amount of antigen or PBS per animal emulsified in Incomplete Freund’s adjuvant (catalog no. F5506, Sigma-Aldrich) at days 14 and 28 after the initial injection. Mouse serum samples separated from blood were collected and stored at − 80 °C on days 0, 14, 28, 42 and 70 for later use.

### Antigenicity evaluation by protein microarrays

Serum reaction against purified recombinant proteins was determined using well-type amine arrays as previously described [30, 31]. For the assays, teflon tapes with holes were pasted on adhesion microscope slides (CITOTEST Scientific, Jiangsu, China) as protein microarrays; 1 μl of rPocDBP-RII (50 μg/μl) was spotted to each well of the arrays and incubated for 2 h at 37 °C. Array slides were blocked with 5% BSA in PBST at 37 °C for 1 h. Serum samples diluted to 1:250 from each of the four blood collection periods or a set of 1:2 serial dilution from 1:250 to 1:32,000 drawn from the third and fourth blood collection periods was added to the chip for 1 h at 37 °C. Pre-immune serum and serum from mice immunized with adjuvant alone served as negative controls. Finally, bound antibodies were visualized using Alexa Fluor 555-labeled Donkey Anti-Mouse IgG (H + L) (1:100 dilution; catalog no. A0460, Beyotime, Shanghai, China), scanned by LuxScanTM 10 K-B Microarray Scanner (catalog no. 100020, CapitalBio); fixed circle approach was used to quantify the mean fluorescence intensity (MFI).

### Mouse spleen cell isolation

Fresh whole mouse spleens were obtained and minced into small pieces with a sterile scissor in petri dish with 10 ml precooled RPMI 1640 (catalog no. 31800, Solarbio, Beijing, China) under a sterile biosafety cabinet. The spleen tissue pieces were gently mashed through the 70-µm strainer using a circular motion with the flat end of the plunger from a syringe until the remnant of the spleen tissue became white. The cell suspension was filtered again and washed with excess RPMI 1640 by centrifuging at 1000 rpm for 5 min at 4 °C. Then, the pellet was resuspended in 3 ml cold RBC lysis buffer for 5 min on ice. Following the incubation, lysis reaction was stopped by adding complete RPMI medium to a final volume of 10 ml. After washing twice, the cells were counted and resuspended to the desired cell concentration with complete RPMI medium.

### Lymphocyte proliferation assays

The proliferation of lymphocyte was determined using the CCK8 assay, as previously reported [30, 32]. Lymphocytes from mice immunized with rPocDBP-RII and PBS (5 × 10^4^ cells/well) were treated with Concanavalin A (Con A: 2 μg/ml) or rPocDBP-RII (10 µg/ml) in 96-well flat-bottom microtiter plates. The complete RPMI 1640 medium and Con A were used as blank and positive controls, respectively. Then, the plates were incubated for 72 h at 37 °C with 5% CO_2_. Cell proliferation was conducted by using the Cell Counting Kit-8 assay, and the stimulation index (SI) was calculated by the following formula: SI = [(OD_450_ of stimulated cells—OD_450_ of unstimulated cells)/OD_450_ of unstimulated cells] × 100%.

### Flow cytometry

To evaluate the response of IFN-γ/IL-10 cytokine-producing T cells and memory T cells upon rPocDBP-RII stimulation, the cell phenotypes were analyzed by flow cytometry (Cytoflex S, Beckman Coulter, CA, USA). Antibodies used in this study were purchased from Biolegend (San Diego, CA, USA) or BD (BD Biosciences, Franklin Lakes, NJ, USA). Evaluation of the response of IFN-γ/IL-10 cytokine-producing cells was done using established protocols [33–35]. Briefly, cells were resuspended in complete RPMI 1640 containing proteins of rPocDBP-RII (10 µg/ml) and plated at 5 × 10^6^ cells/well in 12-well plates. After 18 h, 2 × 10^6^ cells were transferred into 1.5-ml Eppendorf tubes and incubated in the presence of PMA (50 ng/ml), ionomycin (5 µg/ml) and Brefeldin A (10 µg/ml) for 4 h at 37 °C with 5% CO_2_ to stimulate T lymphocytes and block cytokine secretion. Cells were then washed once in PBS and stained with CD3 (FITC), CD4 (APC) and CD8a (PE-Cy7) for 30 min at 4 °C. Cells in each tube were washed again and fixed in 200 μl of 4% paraformaldehyde solution for 20 min at 4 °C. For rPocDBP-RII stimulation for 24 h, the cell surface markers for memory T cells [CD3 (APC-Cy7), CD4 (PE), CD8a (APC), CD44 (FITC), CD62L (PE-Cy7)] were stained in the same way. For intracellular cytokine staining, the cells were then permeabilized with a cell permeabilization kit (eBioscience) according to the manufacturer’s instructions and stained with IFN-γ (PE) and IL-10 (PE) in the dark for 30 min at 4 °C, respectively. Finally, cells were washed and resuspended in flow cytometry staining buffer prior to flow cytometric analysis. Data were analyzed with FlowJo v10 (TreeStar, LaJolla).

### Statistical analysis

GraphPad Prism (version 8.4.2; GraphPad Software, San Diego, CA, USA) and SigmaPlot (Systat Software Inc., San Jose, CA, USA) were used for the statistical analyses and graphing. An unpaired two-tailed Student's t-test was used for the comparison of mean values between samples; *P* < 0.05 was considered significant.

**Fig. 1:**
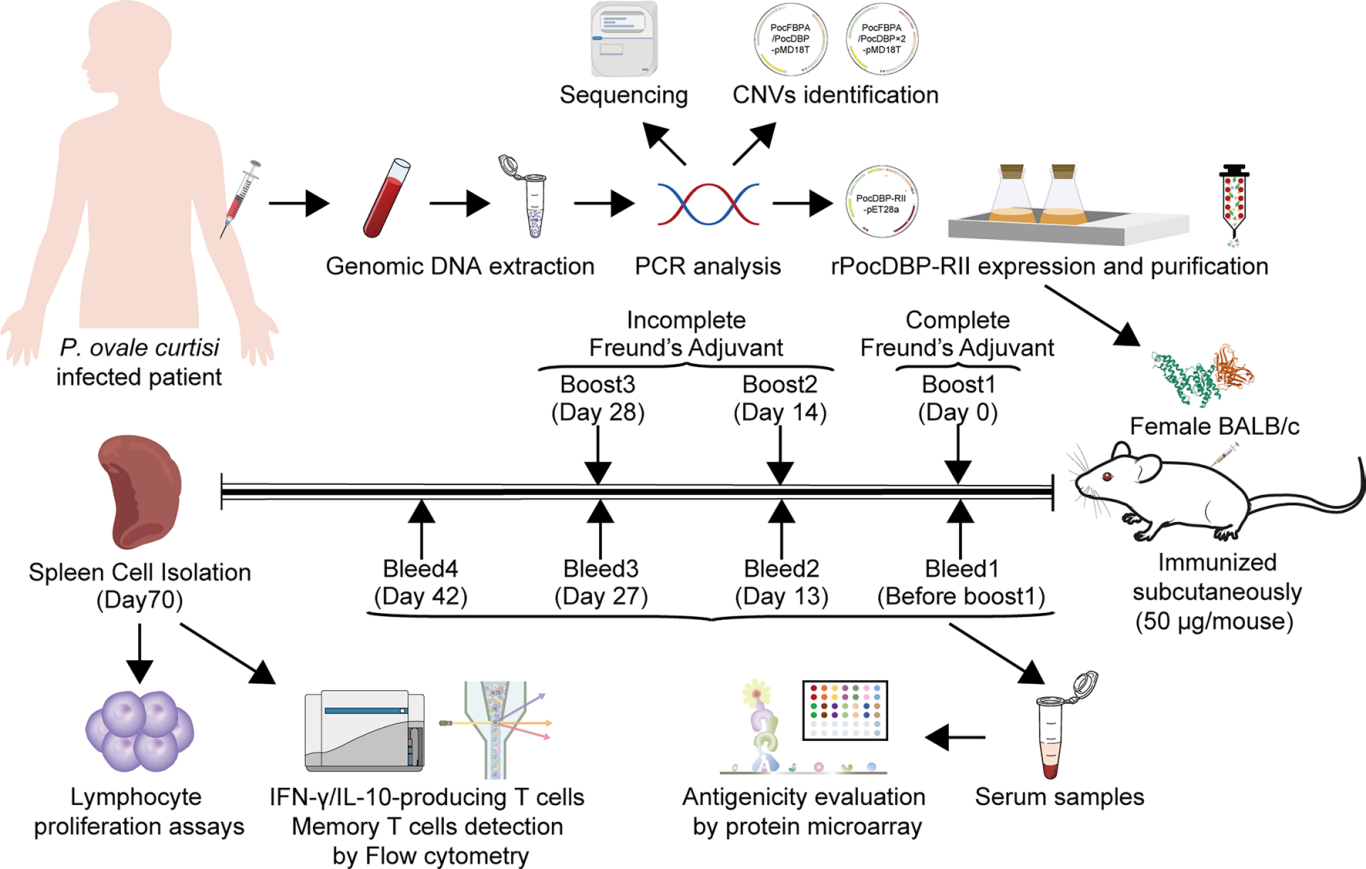
Schematic illustration of the experimental process. Genomic DNA was extracted from blood samples of patients infected with *Plasmodium ovale curtisi*. Genetic polymorphisms and natural selection of *PocDBP-RII* gene were investigated by sequencing and neutral tests. Copy number variation of *PocDBP-RII* gene was studied by qPCR. The proliferation of lymphocytes in mice immunized with rPocDBP-RII was determined using the CCK8 assay. Levels of anti-rPocDBP-RII antibody response in serum and IFN-γ/IL-10-producing T cells in spleens of immunized mice were assessed by protein microarrays and flow cytometry, respectively

The schematic diagram of the whole experimental process is shown in Fig. 1.

## Results

### Nucleotide polymorphism and natural selection of PocDBP-RII

**Fig. 2:**
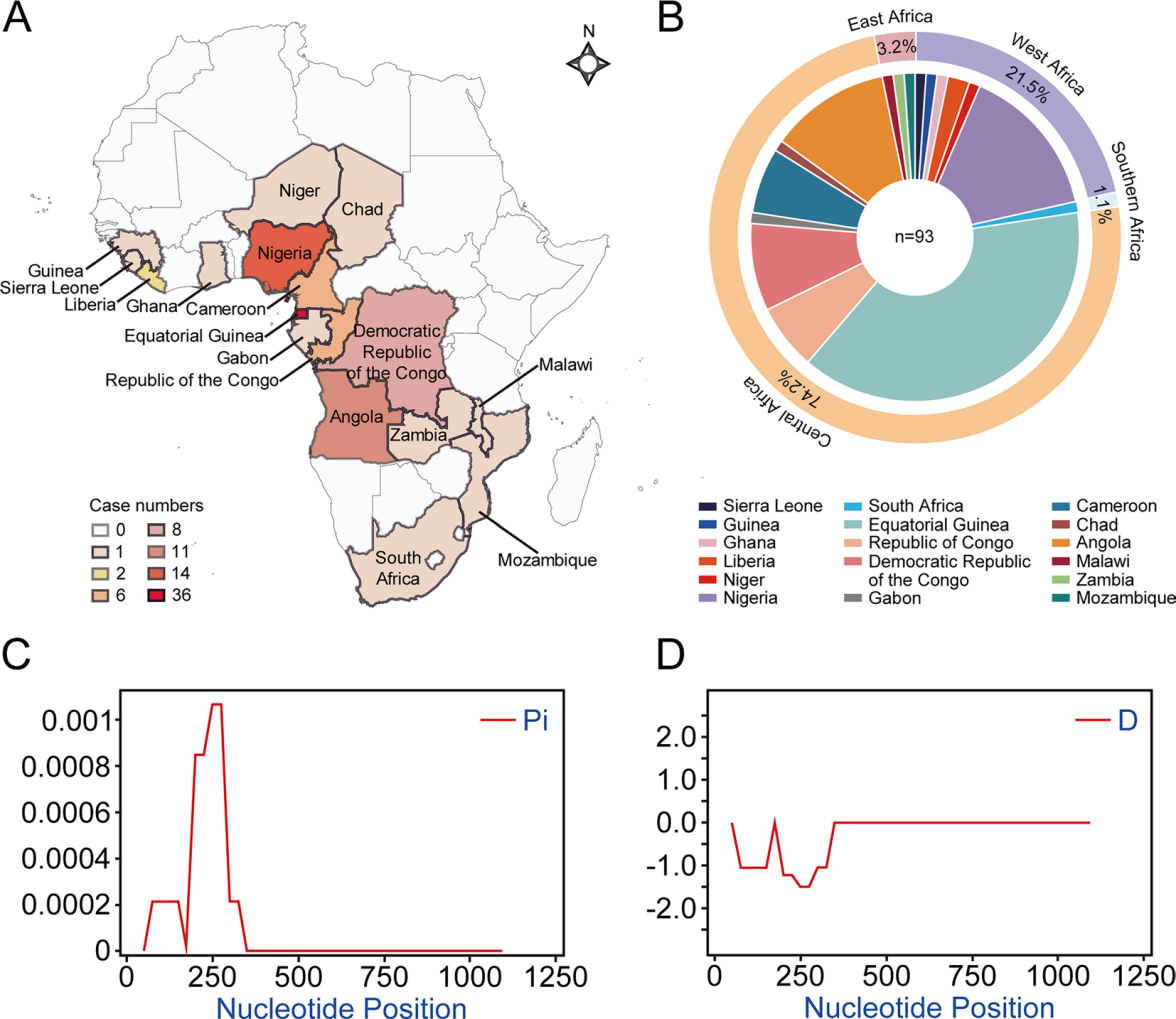
Geographical distribution of sources of the *Plasmodium ovale curtisi* clinical samples used in this study and sliding window plot analyses. **A** A map of Africa showing the countries of origin of *P. ovale curtisi* isolates. **B** The total number of genotyped samples (*n* = 93) and percentage of samples per region. Sliding window plot analyses showing nucleotide diversity (π) **C** and Tajima’s D values **D** of *PocDBP-RII*

To determine the extent of genetic variation in *PocDBP-RII*, 93 *P. ovale curtisi* samples were sequenced for the *PocDBP-RII*, including Angola (*n* = 11), Cameroon (*n* = 6), Chad (*n* = 1), Democratic Republic of the Congo (n = 8), Equatorial Guinea (*n* = 36), Gabon (*n* = 1), Ghana (*n* = 1), Guinea (*n* = 1), Liberia (*n* = 2), Malawi (*n* = 1), Mozambique (*n* = 1), Niger (*n* = 1), Nigeria (*n* = 14), Republic of Congo (*n* = 6), Sierra Leone (*n* = 1), South Africa (*n* = 1) and Zambia (*n* = 1) (Fig. 2A, B). All sequences were aligned and cut to 1143 bp (nt, 526–1668) by MEGA7.0. There were five haplotypes (H) and four SNPs, including three singleton variable sites and one parsimony informative sites. The haplotype diversity (Hd) of *PocDBP-RII* gene was 0.105, and the nucleotide diversity index (*π*) was 0.00011 (Table 2). The *π* value ranging from 0 to 0.00106 was evaluated by a 100-bp sliding window with a 25-bp increment (Fig. 2C), revealing all the polymorphic sites were concentrated in the N-terminal region of *PocDBP-RII* (nt, 601–850).

**Table 2:** Estimates of nucleotide diversity, haplotype diversity and neutrality indices of *PocDBP-RII* domain based on the geographical location

No. of	SNPs	No. of	Diversity ± SD		Tajima’s D	Fu and Li’s	
Samples		Haplotype (H)	Haplotype (Hd)	Nucleotide (π)		D^*^	F^*^
93	4	5	0.105 ± 0.044	0.00011 ± 0.00005	− 1.6812	− 2.63945^a^	− 2.74087^a^

^*^P < 0.05 was considered significant

Neutral test was performed to identify causes of species-specific phenotype. All these five isolates with base mutation showed non-synonymous mutations, containing one A/C polymorphism at nucleotide 629 (K210T), two T/A polymorphisms at nucleotide 751 (F251I), one TT/AA polymorphism at nucleotide 752–753 (F251N) and one C/G polymorphism at nucleotide 813 (N271K) (Table 3). However, neither the ratio of non-synonymous to synonymous substitutions (*dN/dS* or *ω*) nor a dN-dS value can be used to detect positive Darwinian selection, because the differences in a set of conspecific sequences sampled from a single population just represent segregating polymorphisms as opposed to fixed substitutions [36]. Tajima’s D was negative (Fig. 2D) but the *P* value was not significant (Tajima’s D =  − 1.6812, 0.05 < *P* < 0.1), showing that the mutations in *PocDBP-RII* in this study were in accordance with the neutral evolution model. However, Fu and Li’s D^*^ and F^*^ tests rejected a neutral polymorphism occurrence model (Fu and Li’s D^*^  =  − 2.63945, *P* < 0.05, and Fu and Li’s F^*^  =  − 2.74087, *P* < 0.05) (Table 2). Since Fu and Li’s D^*^ and F^*^ tests are very sensitive to recent populations expansions, there are cases where Fu and Li’s D^*^ and F^*^ statistics values are negative and significant and Tajima’s D is near zero. Among the intra-species tests, the partial DNA sequencing and analyses unveiled limited genetic diversity of *PocDBP-RII* and weak evidence for natural selection.

**Table 3:** Prevalence of *PocDBP-RII* gene substitution in 93 parasite isolates

No	Amino acid change	Residue change	Conservative(C) or non-conservative change (NC)	Frequency (%)
1	A629C	K210T	NC	1 (1.08)
2	T751A	F251I	NC	3 (3.23)
3	T752A	I251N	NC	1 (1.08)
4	C813G	N271K	NC	1 (1.08)

### Copy number estimation of PocDBP-RII among collected blood samples

**Fig. 3:**
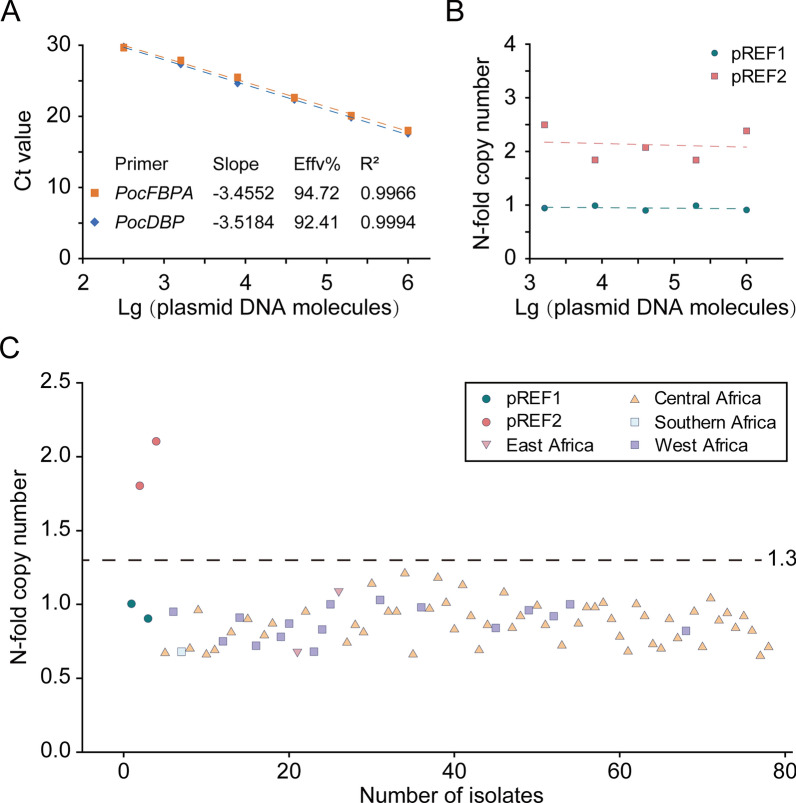
Copy number variation in *PocDBP-RII* gene. **A** Standard curve of qPCR for detection of *PocDBP-RII* and *PocFBPA* to calculate relative amplification efficiency (Effv%). The horizontal axis represents the Lg of DNA molecules added to the qPCR reaction system at each dilution, and the vertical axis is Ct values. The absolute value of the slope was close to zero (*m* = 0.0632), confirming that the amplification efficiencies of *PocDBP-RII* and *PocFBPA* were similar. **B** Plots of the Lg of DNA molecules versus N-fold copy number were made to reflect the relative efficiency and *PocDBP-RII* copy number in the two reference plasmids. **C** Scatter plots depicting the estimation of *PocDBP-RII* N-fold copy number in *Plasmodium ovale curtisi* isolates from different regions of Africa (*n* = 74)

To detect CNVs in *PocDBP-RII* gene, the 2^−∆∆Ct^ method of relative quantification using real-time quantitative PCR with SYBR Green detection was adapted to calculate copy numbers of *PocDBP-RII* [37]. The housekeeping gene *PocFBPA* (PlasmoDB, PocGH01_12070000), which is homologous to *P. vivax aldolase* [8] (PlasmoDB, PVX_118255), was selected as the reference. To assure the validity of ∆∆Ct calculation [∆∆Ct = (Ct _DBP_-Ct _FBPA_) _samples_—(Ct _DBP_-Ct _FBPA_) _pREF1_], amplification efficiency of qPCR was tested. A standard curve was generated using a series of fivefold diluted plasmid DNA samples as template (Fig. 3A), where qPCR efficiencies [Efficiency = (10^−1/slope^–1) × 100%] of reference gene and target gene were similar (*m* < 0.1) and > 90%, meeting the requirements of recommended amplification efficiency [38]. Furthermore, N-fold copy number (2^−∆∆Ct^) observed in pREF2 was obviously twice the number in pREF1 at each dilution (Fig. 3B), confirming that the 2^−∆∆Ct^ calculation could be applied.

Of all 93 isolates, 2 of 3 from East Africa, 16 of 20 from West Africa, 1 of 1 from Southern Africa and 55 of 69 from Central Africa were successfully determined. Our estimates of *PocDBP-RII* CN for these isolates ranged from 0.7 to 1.2 (Fig. 3C). In no cases was N-fold > 1.3, indicating that all the isolates carried one copy of the *PocDBP-RII* gene. Nineteen samples that did not meet the criteria (N-fold > 0.7) used in the methodology were excluded from the analysis. The failure of the CN detection in these samples may have been caused by the low-grade parasitemia resulting in too little *P. ovale curtisi* gDNA extracted for qPCR. This situation was also present in the process of calculating the N-fold copy number of pREF1 and pREF2, where the N-fold copy numbers exhibited great instability when the DNA molecule was < 320 (data not shown). As *PocDBP-RII* is evolutionarily stable and free of CNV, enabling it to meet the primary requirement for developing a valuable antigen as vaccines, the next step was to ensure its antigenicity and immunogenicity.

### Expression and purification of the rPocDBP-RII

**Fig. 4:**
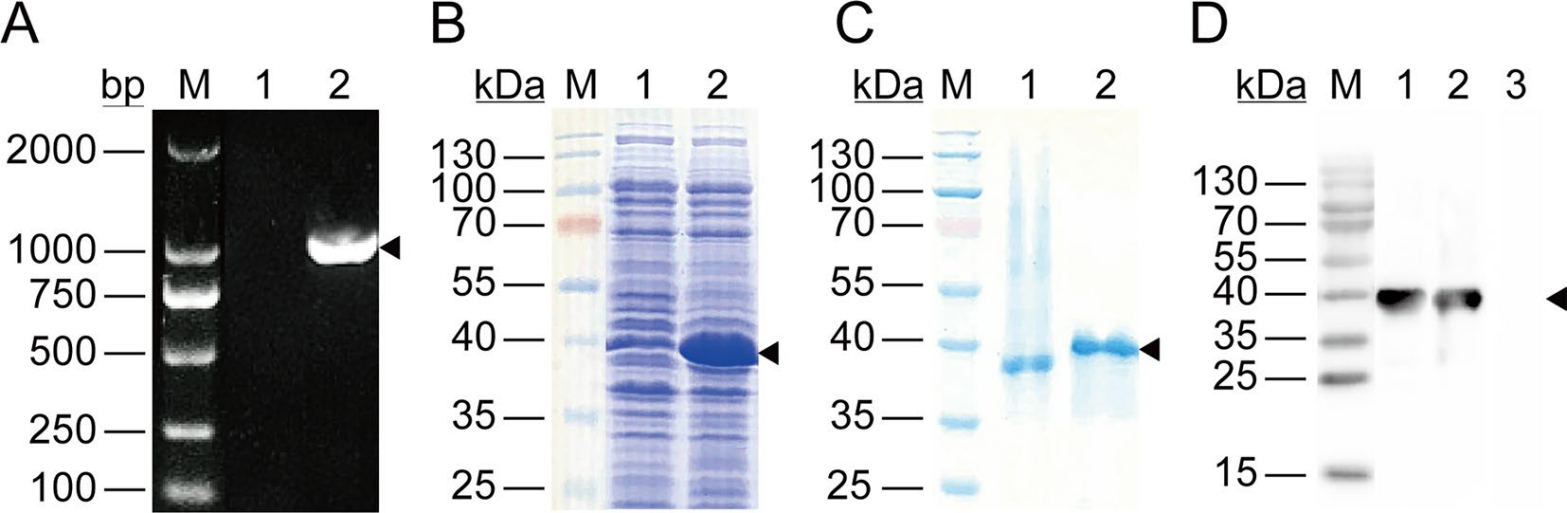
Expression and purification of rPocDBP-RII and detection of anti-rPocDBP-RII antibody in sera of immunized mice. **A** Polymerase chain reaction (PCR) amplification for *PocDBP-RII* (arrowhead: a 975-bp product). M: marker; lane 1: no template control; lane 2: DNA extracted from the plasma of parasite-infected patient. **B** SDS-PAGE analysis of rPocDBP-RII (arrowhead: a 39-kDa band) after induction with IPTG. M: protein marker; lane 1: cell homogenates of *E. coli* Rosetta/pET28a-PocDBP-RII without IPTG induction; lane 2: cell homogenates after IPTG induction for 6 h. **C** Coomassie blue-stained SDS-PAGE gel of eluted, purified and concentrated rPocDBP-RII before and after treatment with dithiothreitol (DTT). M: protein marker; lane 1: refolded rPocDBP-RII (DTT-); lane 2: denatured rPocDBP-RII (DTT +) which migrated as a single banding pattern on SDS-PAGE at the expected size of 39 kDa. **D** Western blot analysis of rPocDBP-RII using an anti-HIS rabbit monoclonal antibody (lane 1) or antibodies from the serum of mice immunized with rPocDBP-RII (lane 2) or PBS (lane 3)

The PCR products of *PocDBP-RII* were successfully amplified (Fig. 4A) and cloned into a pET28a vector. The resulting plasmid was transformed into *E. coli* strain Rosetta (DE3) cells and induced with IPTG. Protein expression was analyzed by SDS-PAGE and then stained with Coomassie brilliant blue (Fig. 4B). The rPocDBP-RII was purified from inclusion bodies, and the refolded protein showed different mobilities under reducing (DTT +) and non-reducing conditions (DTT-), indicating that the disulfide bonds had been correctly formed (Fig. 4C). Bicinchoninic acid (BCA) assay was used to create a standard curve, and the concentration of purified rPocDBP-RII obtained was estimated to be 1 mg/ml. Horseradish peroxide (HRP) conjugated anti-His antibody was used in western blot to detect protein expression, and sera from rPocDBP-RII-immunized mice or PBS-immunized mice were employed to study the antibody specificity of rPocDBP-RII-immunized mice (Fig. 4D). As a result, rPocDBP-RII-immunized mouse serum were demonstrated to specifically recognize rPocDBP-RII protein compared with lack of recognition in PBS-immunized mouse serum (negative control).

### Humoral immune response induced by rPocDBP-RII in mice

**Fig. 5:**
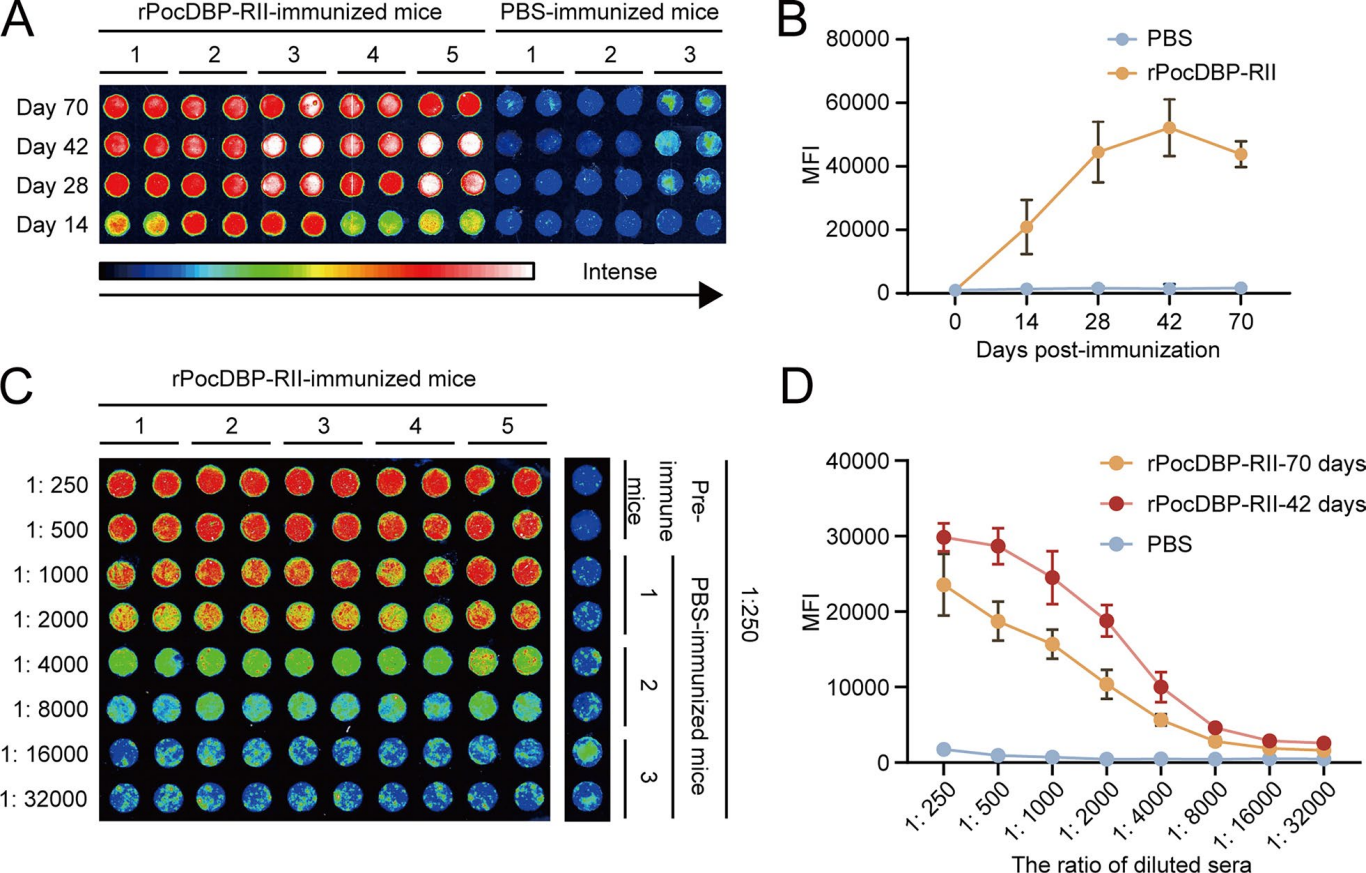
Humoral immune responses in rPocDBP-RII‑immunized mice. **A** Levels of total IgG in mouse serum against rPocDBP-RII protein (*n* = 5) or PBS (*n* = 3) were measured by protein arrays on days 0, 14, 28, 42 and 70 of post immunization. False color image of an example array shows signal intensity; a white or red spot is more intense than the blue ones. The number at the top of the picture represents different mice. **B** Quantification of the relative spot intensities of duplicates. Higher concentrations of antibody in the serum produce stronger mean fluorescence intensity (MFI). **C** Serum from immunized mice was arrayed in duplicate in a series of two-fold dilutions (from 1: 250 to 1: 32,000). **D** Linearity and replicability of this data is shown in the graph

To evaluate the antigenicity of rPocDBP-RII, the average serum IgG titers of antigen-specific antibodies in rPocDBP-RII‑immunized and PBS‑immunized mice were assessed by protein microarrays. Pre-immunization blood samples of mice were obtained to establish a baseline pre-immune level and normalize median fluorescence intensity on different protein microarrays. The results indicate that, compared to PBS-immunized mice, antibody levels of rPocDBP-RII gradually increased from the primary immunization until approximately 2 weeks after the last immunization (day 42) and began to drop in the following month but still maintained a high level (Fig. 5A, B). In addition, after three consecutive immunizations with rPocDBP-RII, serum samples from immunized mice diluted from 1:250 to 1:32,000 on days 42 and 70 after initial immunization showed a similar tendency of reactivity to rPocDBP-RII, and the antibody titer to target antigen is 1:16,000 (Fig. 5C, D).

### IFN-γ-producing T cells play a role in the immune response to rPocDBP-RII antigen

**Fig. 6:**
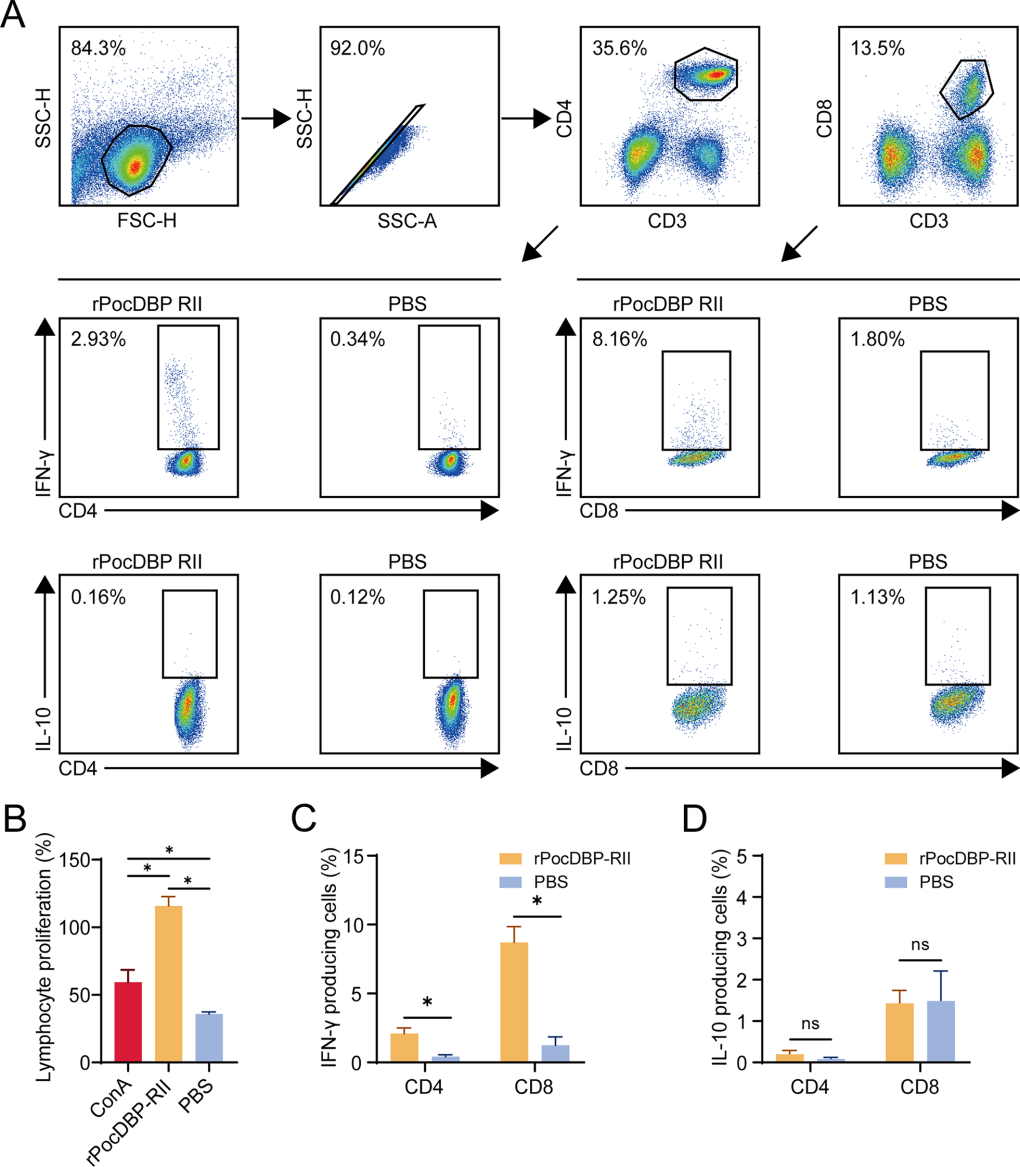
IFN-γ/IL-10-producing T cell responses in rPocDBP-RII‑immunized mice. **A** Flow cytometry gating strategy for IFN-γ/IL-10-producing T cells upon rPocDBP-RII antigen. **B** Lymphoproliferative activity of splenocytes isolated from experimental group and PBS group, concanavalin A (Con A) worked as a positive control. Measurement of IFN-γ-producing **C** and IL-10-producing **D** CD4^+^ and CD8^+^ T cell levels in mice by flow cytometry. The data are shown as the mean ± SD. *P* values were calculated using Student’s t‑test. Significant differences between groups are denoted on the graph: * *P* < 0.05, nonsignificant (*ns*) differences are indicated (*P* > 0.05)

Lymphocyte proliferation assay was used to evaluate the immunogenicity of rPocDBP-RII in induction of antigen-specific T cell responses. Compared with the PBS-immunized group, splenocytes of rPocDBP-RII-immunized mice had stronger ability to divide in response to the stimulation of rPocDBP-RII. In addition, the rPocDBP-RII protein induced a more aggressive proliferative response in splenocytes than ConA (positive control) (Fig. 6B). To assess the function of rPocDBP-RII-specific T cells, the proportion of IFN-γ/IL-10 cytokine-producing cells upon rPocDBP-RII stimulation was analyzed by intracellular cytokine staining (Fig. 6A). By stimulating the splenocytes of immunized mice in vitro, the level of IFN-γ-producing T cells was found to be significantly higher in the rPocDBP-RII-immunized group (CD4^+^, 2.09 ± 0.42%; CD8^+^, 8.7% ± 1.15%) than in the PBS-immunized mice (CD4^+^, 0.42 ± 0.13%, *P* < 0.05; CD8^+^, 1.24 ± 0.62%, *P* < 0.05) (Fig. 6C). In contrast, stimulation with rPocDBP-RII could not increase the magnitude of IL-10-producing CD4^+^ T cells (rPocDBP-RII, 0.2 ± 0.09%; PBS, 0.08 ± 0.04%, *P* > 0.05) or CD8^+^ T cells (rPocDBP-RII, 1.43 ± 0.32%; PBS, 1.49 ± 0.72%, *P* > 0.05) (Fig. 6D). These data suggest that immunization with rPocDBP-RII was capable of inducing IFN-γ-producing T cells and that cellular immunity might also be involved in rPocDBP-RII-mediated protection against *P. ovale curtisi* infection.

### Memory T cells response specific to the rPocDBP-RII

**Fig. 7:**
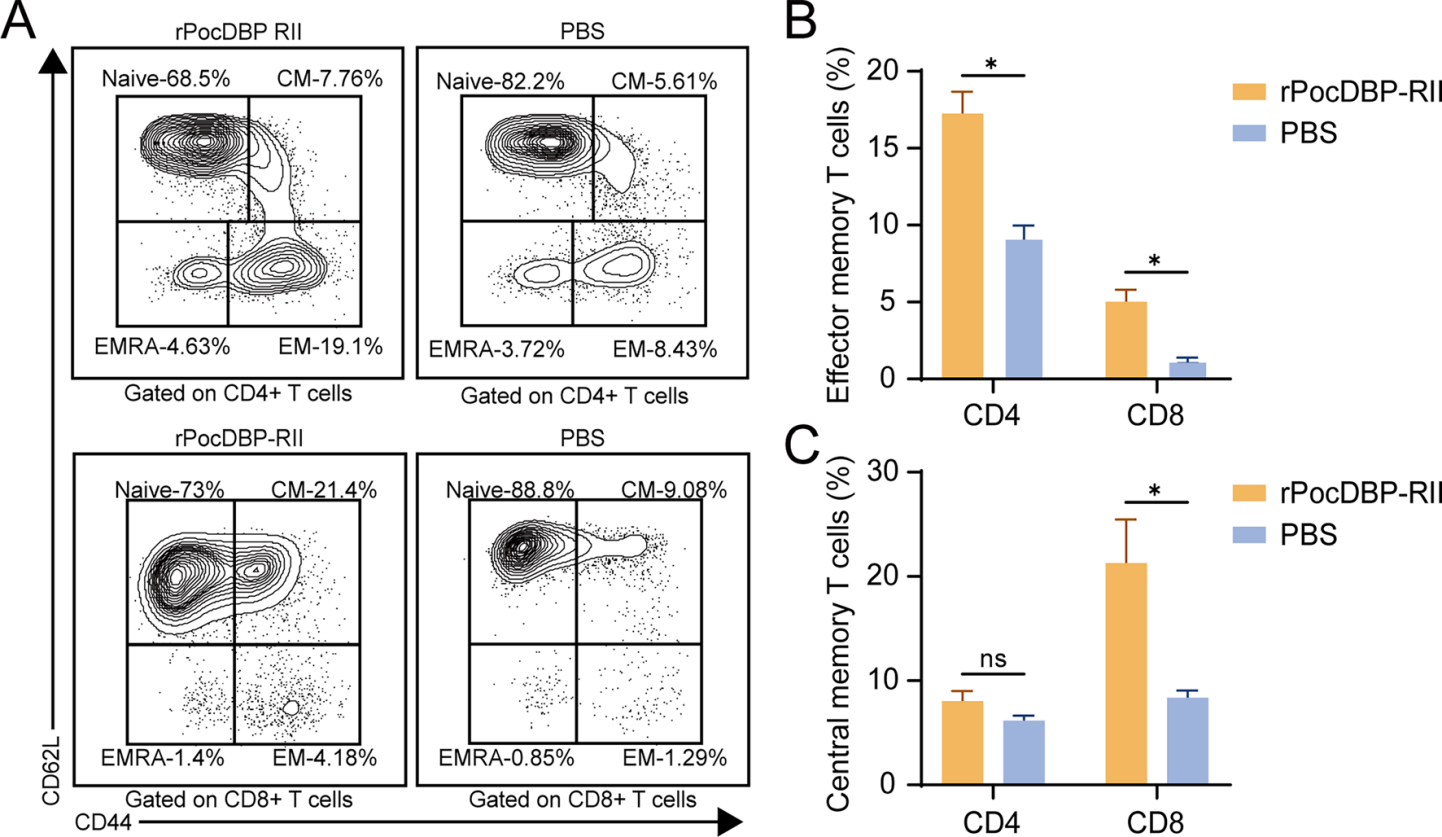
Frequency of memory T cells in response to the stimulation of rPocDBP-RII antigen using multiparameter flow cytometry. **A** The representative gating strategy to identify T naïve cells (T_naïve_, CD44^int/low^ CD62L^+^), T central memory cells (T_CM_, CD44^high^ CD62L^+^), T effector memory cells (T_EM_, CD44^high^ CD62L^−^) and T terminally differentiated cells (T_EMRA_, CD44^int/low^ CD62L^−^). The contour plots above depict CD4^+^ T cells, and the contour plots below show CD8^+^ T cells. Percentage comparison of T_EM_ (**B**) and T_CM_ (**C**) in CD4^+^ and CD8^+^ T cells after stimulating the splenocytes of rPocDBP-RII‑immunized and PBS‑immunized mice by rPocDBP-RII in vitro

As IFN-γ-producing effector/effector memory cells play a key role in the induction and maintenance of long-term protection against malaria mediated by CD4^+^ T cells or CD8^+^ T cells [39], gated CD4^+^ and CD8^+^ splenocytes were analyzed for expression of CD62L and CD44. CD4^+^ and CD8^+^ T cells in the splenocytes of mice can be divided into four naive/memory T cell subsets [40]: CD44^int/low^CD62L^+^ naive, CD44^high^CD62L^+^ CM, CD44^high^CD62L^−^ EM and CD44^int/low^CD62L^−^ EMRA (Fig. 7A). Both the phenotypic pattern of CD4^+^ and CD8^+^ T cells in rPocDBP-RII-immunized group showed a higher and significant percentage of T_EM_ (Fig. 7B), whereas T_CM_ showed a significant difference only in the subset of antigen-specific CD8^+^ T cells (Fig. 7C). Furthermore, our study showed that rPocDBP-RII could induce a higher proportion of T_EM_ in CD4^+^ T cells (CD4^+^, 17.26% ± 1.41%; CD8^+^,5.03% ± 0.78%) and a higher proportion of T_CM_ in CD8^+^ T cells (CD4^+^, 8.03% ± 0.98%; CD8^+^, 21.28% ± 4.18%). In general, CD4^+^CD44^high^CD62L^−^ T cells and CD8^+^CD44^high^CD62L^+^ T cells activated by stimulation of rPocDBP-RII appeared to predominate.

## Discussion

Recently, non-*falciparum* and non-*vivax* malaria have become the focus of the scientific community with changing patterns of malaria transmission and good control of *P. falciparum* [41]. Here, we show that *PocDBP-RII* had lower nucleotide diversity (*π* = 0.00011) compared to other vaccine candidates for *P. vivax*, such as *PvEBP*, *PvDBP-RII, PvAMA1* and *PvMSP3α* (*π* = 0.019) [42, 43]. Protein sequence analysis revealed that there were three polymorphic residue sites in *PocDBP-RII*, of which amino acid change at position 251 was trimorphic (F251I, F251N), occurring with a frequency of 3.2%. Interestingly, a clustal alignment of homologous sequences revealed that amino acid PvDBP-RII-F261, which is homologous to *PocDBP-RII*-F251, was also previously reported to have polymorphism in *P. vivax* isolates from Malaysia [44]. The significance of this mutation needs to be experimentally assessed for further confirmation.

In neutral evolutionary tests, it is somewhat paradoxical that Tajima’s D and Fu and Li’s D* and F* both revealed negative values, but only Fu and Li’s D* and F* reached statistical significance. A similar data pattern was observed in *PvRBP1a-ecto* [42]. The genetic differentiation (F_ST_) estimation and haplotype network analysis indicated that there was no differentiation level in the population considered (data unshown). However, if a population expansion produced an excess of rare alleles, then low nucleotide diversity and high haplotypes would coexist [45]. Yet the fact that we found only five haplotypes cannot account for the presence of natural selection. Genetic diversity analysis of *PocDBP-RII* indicated that this region with no amplification is conserved to be an attractive target region for vaccine development. However, larger global samples and more powerful techniques will be needed in future research to support our findings.

Antigens with low genetic diversity and conserved amino acid sites can serve as potential targets for protective immune responses and vaccine candidates [46]. The contribution of potent neutralizing immune responses and protective cellular immunity to the prevention of malaria has recently been examined in the context of vaccine development [47–49]. Serological response indicated that rPocDBP-RII may be the target of immunologically active antibodies. Though antibody titers waned after 2 months of immunization, the antibody level obtained from immunized mice was still high. Besides, suitable adjuvants are sufficiently effective in inducing high antibody titers [50], and the longevity and composition of the cellular immune response seems to deserve more attention. An effective vaccine will initiate a humoral immune response for the development of malarial antibodies through B cells and a cellular immune response through the production of antigen-specific lymphocytes by T cells [51]. A sufficient amount of the plasmodium parasite-specific antibodies and CD4^+^ and CD8^+^ T cells can mediate protective blood-stage immunity [52]. Our results suggested that immunization with rPocDBP-RII was capable of inducing IFN-γ-producing T cells, rather than IL-10-producing T cells, to participate in rPocDBP-RII-mediated cellular immunity against *P. ovale curtisi* infection. Several studies have repeatedly shown that IFN-γ is essential for parasite clearance and protective efficacy [53, 55]. In malaria infection, IL-10 is a potent suppressor of several pro-inflammatory cytokines such as IFN-γ, making it detrimental to the cell-mediated immune response [56, 57].

Although IFN-γ can be secreted by many cells of the immune system, the production of long-lived malaria-specific IFN-γ memory responses is primarily driven by memory T cells [58, 60]. This study confirms that both CD4^+^ T cells and CD8^+^ T cells were crucial producers of IFN-γ, but CD8^+^ T cells showed a higher rate of T_CM_ while CD4^+^ T cells displayed a higher proportion of T_EM_. The result was inconsistent with the conclusion that CD4^+^ T cells were the major source of IFN-γ in response to PvMSP1P-19 [38], because though most spleen IFN-γ may be produced by CD4^+^ T cells in the early stage of infection, at the late stage IFN-γ from CD8^+^ T cells could be enhanced because of compensation by other cell types [19]. The boost to CD4^+^ T_EM_ associated with antigen persistence may be caused by driving their proliferation and by programming production of T_EM_ from T_CM_ [21, 58]. All in all, CD44^high^ memory T cells may contain a large proportion of effector cells producing IFN-γ, which may have contributed to the enhanced protection. Furthermore, it should be noted that this paper concentrates largely upon the immune response of T cells; however, it will often be desirable to measure at the same time as memory B cells (MBCs) and long-lived plasma cells (LLPCs) to develop long-lasting and effective vaccine candidate [35, 61]. In conclusion, this study investigated new information on the suitability of rPocDBP-RII as a novel vaccine candidate for *P. ovale curtisi*.

## Conclusions

This study confirms that the *PocDBP-RII* gene, which has low genetic diversity and weak evidence of natural selection, is conserved among *P. ovale curtisi* parasites collected from African isolates. Not only that, but the rPocDBP-RII antigen has been observed to efficiently provoke and uphold both humoral and T-cell-derived IFN-γ-dependent cellular responses in mice. These findings suggest that rPocDBP-RII could serve as a potential vaccine candidate, but it is imperative that future studies be conducted to further gauge the efficacy of rPocDBP-RII.

## Data Availability

All data generated or analyzed during this study are included in this published article.

## Abbreviations

PocDBP-RII: *Plasmodium ovale curtisi* Duffy binding protein domain region II
PCR: Polymerase chain reaction
nt: Nucleotide
SDS-PAGE: Sodium dodecyl sulfate–polyacrylamide gel electrophoresis
PBST: Phosphate‑buffered saline with Tween-20
